# Long non-coding RNA linc00665 promotes lung adenocarcinoma progression and functions as ceRNA to regulate AKR1B10-ERK signaling by sponging miR-98

**DOI:** 10.1038/s41419-019-1361-3

**Published:** 2019-01-28

**Authors:** Zhuangzhuang Cong, Yifei Diao, Yang Xu, Xiaokun Li, Zhisheng Jiang, Chenye Shao, Saiguang Ji, Yi Shen, Wei De, Yong Qiang

**Affiliations:** 1 Department of Cardiothoracic Surgery, Jinling Hospital, Medical School of Nanjing University, 210000 Nanjing, China; 2 Department of Cardiothoracic Surgery, Jinling Hospital, Southeast University, 210000 Nanjing, China; 3 Department of Cardiothoracic Surgery, Jinling Hospital, Nanjing Medical University, 210000 Nanjing, China; 4 Department of Cardiothoracic Surgery, Jinling Hospital, Bengbu Medical College, 233030 Anhui, China; 5 Department of Biochemistry and Molecular Biology, Nanjing Medical University, 210000 Nanjing, China

## Abstract

Long non-coding RNAs (lncRNAs) are frequently dysregulated in multiple malignancies, demonstrating their potential oncogenic or tumor-suppressive roles in tumorigenesis. Herein, we reported the identification of a novel lncRNA, linc00665 (ENST00000590622), which was markedly upregulated in lung adenocarcinoma (LUAD) tissues and might serve as an independent predictor for poor prognosis. Functional assays indicated that linc00665 reinforced LUAD cell proliferation and metastasis in vitro and in vivo. Mechanistically, transcription factor SP1 induced the transcription of linc00665 in LUAD cells, which exerted its oncogenic role by functioning as competing endogenous RNA (ceRNA) for miR-98 and subsequently activating downstream AKR1B10-ERK signaling pathway. Together, our study elucidates oncogenic roles of linc00665–miR98–AKR1B10 axis in LUAD tumorigenesis, which may serve as potential diagnostic biomarkers and therapeutic targets.

## Introduction

Lung cancer remains the most common incident cancer in China and the leading cause of cancer death worldwide^1,2^. Non-small cell lung cancer (NSCLC) accounts for 85% of all lung carcinomas, and lung adenocarcinoma (LUAD) contributes to the most common histological subtype. Despite the advances in diagnostic and therapeutic strategies, clinical outcomes of LUAD have not substantially improved, generally attributed to late diagnosis and tumor metastasis. Thus, deciphering the molecular mechanisms underlying the initiation and progression of LUAD is a priority to identify novel diagnostic biomarkers and therapeutic targets.

It has been estimated that the human genome is actively transcribed; however, only 2% of the transcripts encode proteins^3^. The vast majority of the transcripts are termed as non-coding RNAs, including microRNAs and long non-coding RNAs (lncRNAs). LncRNAs, commonly defined as transcripts longer than 200 nucleotides with limited or no protein-coding capacity, participate in diverse cellular, physiological, and pathological processes, by acting through a broad array of mechanisms^4–6^. Specifically, the competing endogenous RNA (ceRNA) hypothesis was proposed to describe lncRNA–microRNA–mRNA crosstalk. In this case, lncRNAs may function as ceRNAs to sponge certain microRNAs hence relieving repression of target mRNAs at a post-transcriptional level^7–10^. Moreover, lncRNAs are frequently dysregulated in multiple malignancies, including lung cancer, demonstrating their potential oncogenic or tumor-suppressive roles in tumorigenesis^5,6,11,12^.

In the present study, we identified a novel lncRNA linc00665 (ENST00000590622, NR_038278), which was markedly upregulated in LUAD tissues and might serve as an independent predictor for recurrence-free survival of LUAD patients. To the best of our knowledge, the biological roles of linc00665 in cancer have not been characterized previously. Thus, we further explored the impact of linc00665 on aggressive phenotypes of LUAD cell lines in vitro and in vivo. Moreover, mechanistic analysis revealed that linc00665 functioned as a miRNA sponge to positively regulate the expression of AKR1B10 through binding miR-98, thereby facilitating LUAD progression. Together, our study elucidates oncogenic roles of linc00665–miR98–AKR1B10 axis in LUAD tumorigenesis, which may serve as potential diagnostic biomarkers and therapeutic targets in LUAD.

## Materials and methods

### Patients and clinical samples

A total of 80 LUAD tissues and their pair-matched adjacent normal tissues were obtained from patients who underwent lobectomy at Jinling Hospital during January 2012 to December 2013. The pathological diagnoses were confirmed postoperatively. The fresh tissues were snap frozen in liquid nitrogen immediately after extraction and stored at –80 ℃. None of the patients received preoperative chemotherapy or radiotherapy. Histopathologic features of tumors were defined according to the 8th edition of American Joint Committee on Cancer (AJCC) staging system. Written informed consent was obtained from all patients, and protocols for this study were approved by the Ethics Committee of Jinling Hospital, Medical School of Nanjing University.

### Cell lines and culture

The NSCLC cell lines (A549, H1299, H1650, H520, SPCA-1, and SK-MES-1), human bronchial epithelial cell (16HBE) and HEK-293T were purchased from Shanghai Institute of Biochemistry and Cell Biology (Shanghai, China). HEK-293T were maintained in Dulbecco’s modified Eagle’s medium (Gibco, USA), and other cells were cultured in RPMI-1640 medium (Gibco, USA), supplemented with 10% fetal bovine serum (FBS, Gibco, USA), in a humidified incubator at 37 °C with 5% CO_2_.

### Cell transfection

Linc00665 and SP1 complementary DNA was synthesized and cloned into the expression vector pcDNA3.1(+). The small interfering RNAs (siRNAs) targeting linc00665, AKR1B10 and SP1, miR-98 mimics and inhibitors and their negative controls were designed and synthesized by GenePharma (Shanghai, China). Transient transfection of siRNA, miRNA or plasmid was performed by using a standard protocol from the Lipofectamine 2000 (Invitrogen, USA). The short hairpin RNA (shRNA) targeting linc00665 and negative control shRNA were ligated into pLKO.1 lentiviral vector, and were stably transfected into A549 cells using a lentiviral gene delivery system as previously described^13^. The sequences of siRNA and shRNA were listed in Supplementary Table 1.

### Quantitative real-time PCR (qRT-PCR)

Total RNA was extracted from tissues and cells using RNeasy Mini Kit (Qiagen, USA) and reversely transcribed using PrimeScript RT reagent kit (Takara, Japan). Mature miR-98 was reversely transcribed using Bulge-Loop miRNA qRT-PCR Starter Kit (RiboBio, China)^14^. Quantitative PCR was then performed using PowerUp SYBR Green Master Mix (Applied Biosystems, USA), and each PCR amplification was performed in triplicate. Primer sequences were listed in Supplementary Table 2. Bulge-Loop miRNA primers were purchased from RiboBio. Data were analyzed applying the 2^−ΔΔCt^ method, normalized to glyceraldehyde 3-phosphate dehydrogenase (GAPDH).

### Western blot

Total cell lysates were extracted from A549 and H1299 cells. Protein samples (30 μg) were separated by 6–12% Sodium Dodecyl Sulphate - PolyAcrylamide Gel Electrophoresis (SDS-PAGE) gels and transferred onto Polyvinylidene Fluoride (PVDF) membranes. The membranes were blocked with 5% skimmed milk and then incubated overnight at 4 °C with primary antibodies: anti-E-cadherin (1:2000, Abcam, ab1416, USA), anti-Vimentin (1:2000, Abcam, ab92547, USA), anti-N-cadherin (1:2000, Abcam, ab98952, USA), anti-Bcl-2 (1:1000, Santa Cruz, sc-7382, USA), anti-cleaved-Caspase-3 (1:1000, Abcam, ab13847, USA), anti-Bax (1:2000, Abcam, ab32503, USA), anti-Extracellular signal-Regulated Kinase (ERK)1/2 (1:1000, Abcam, ab54230, USA), anti-phospho-ERK (p-ERK)1/2 (1:1000, Cell Signaling, #9101, USA), anti-MMP2 (1:1000, Abcam, ab37150, USA), anti-AKR1B10 (1:1500, Abcam, ab139685, USA), anti-SP1 (1:2000, Abcam, ab13370, USA), anti-GAPDH (1:2000, Abcam, ab9485, USA). Bands were developed following incubation with horseradish peroxidase-conjugated secondary antibodies.

### Subcellular fractionation

The nuclear and cytosolic fractions of A549 or H1299 cells were separated using the PARIS Kit (Invitrogen, USA) according to the manufacturer’s instructions. RNA was extracted from both fractions. qRT-PCR was then performed using GAPDH as the cytosolic control, and U6 as the nuclear control.

### Fluorescence in situ hybridization (FISH)

Linc00665, 18S, and U6 probes were obtained from RiboBio (Guangzhou, China). RNA FISH was performed with A549 cells using Fluorescent In Situ Hybridization Kit (RiboBio) following the manufacturer’s instructions.

### CCK-8 proliferation assay

Cells were seeded into 96-well plates at a density of 2 × 10^3^/well. According to the manufacturer’s protocol, 10 μl of CCK-8 (Dojindo, Japan) was added to each well and incubated for 2 h at each time point (6, 24, 48, 72 h). After incubation, cellular viability was determined by measuring the absorbance at 450 nm.

### Colony formation assay

Cells were seeded into six-well plates at a density of 400/well. Ten days later, colonies were fixed with methanol and stained with crystal violet. Colonies containing at least 50 cells were scored.

### Wound-healing assay

Cells were incubated in six-well plates until cultures reached 80–90% confluency. The cell monolayer was scratched in a straight line with a 200 μl pipette tip, washed with phosphate-buffered saline (PBS), and then cultured in medium containing 1% FBS. Images were captured at each time point (0, 24, 48 h).

### Transwell migration and invasion assays

Transwell assays were carried out using 24-well plates with 8 μm pore-size transwell inserts (Millipore, USA). A549 or H1299 cells were counted and resuspended in serum-free medium. For migration assays, 300 μl serum-free medium containing 2.5 × 10^4^ cells were added to the upper chamber, whereas 1 × 10^5^ cells were added to the upper chamber pre-coated with 1:8 diluted Matrigel (BD Biosciences, USA) for invasion assays. The lower chambers were filled with 700 μl medium containing 10% FBS. After incubation for 24 h, cells were fixed with methanol, and the adherent cells on the bottom surface of the insert were stained with crystal violet. Images were taken and cells were counted at ×200 magnification under a light microscope.

### Flow cytometric analysis

To assess the effect of linc00665 downregulation on cell cycle distribution and apoptosis, flow cytometry assays were conducted. Cells were transfected with si-Linc00665 or control siRNA, and were collected 48 h after transfection. According to the manufacturer’s instructions, cell cycle distribution was achieved using PI/RNase Staining Buffer (BD Pharmingen, USA), and apoptosis assay was performed using FITC Annexin V Apoptosis Detection Kit I (BD Pharmingen, USA). Stained cells were analyzed by FACSCalibur Flow Cytometer (BD Biosciences, USA).

### Chromatin immunoprecipitation assay

Chromatin immunoprecipitation (ChIP) assays were performed using the EZ ChIP Kit (Millipore, USA), according to the manufacturer’s instructions. Briefly, cell lysates were sonicated to shear DNA into 200–1000 bp fragments and immunoprecipitated with anti-SP1 antibody (Abcam, ab13370, USA) or normal rabbit IgG as negative control. Immunoprecipitated DNA was then analyzed using qRT-PCR, and quantified as a percentage relative to the input DNA by the equation 2^[Ct(Input)-Ct(ChIP)]^. The primers used were as follows: forward 5ʹ-CCTGGAAACGGACTGTCTGCC-3ʹ, reverse 5ʹ-AGACCAACCTTGCGCCAGGC-3ʹ (product length 107 bp).

### In vivo tumor formation

The animal experiments were conducted in accordance with the Guide for the Care and Use of Laboratory Animals published by the US National Institutes of Health, and the protocol was approved by the Animal Care and Use Committee of Jinling Hospital, Medical School of Nanjing University. A549 cells (3 × 10^6^) stably transfected with shRNA-Linc00665 or shRNA-NC were suspended in 100 μl PBS and subcutaneously injected into the right flank of 4-week-old female BALB/c athymic nude mice (*n* = 10 per group). Tumor volumes were measured as length × width^2^ × 0.5 in mice on a weekly basis. Eight weeks later, the mice were sacrificed and the tumors were excised and snap frozen for further study. The lungs were also removed and visible tumor nods on the lung surface were counted.

### Immunohistochemistry

Immunohistochemistry staining was performed on paraffin-embedded tumor sections from nude mice, as described in our previous study^13^. Primary antibody anti-Ki-67 (1:400, Abcam, ab15580, USA) was used to evaluate the proliferation, and the staining positivity was quantified in three different high-power fields of each section.

### Transcriptome sequencing

Total RNA was isolated from the A549 cells with linc00665 knockdown and control A549 cells. Transcriptome sequencing was conducted using Illumina HiSeq™ 2000 by BGI (The Beijing Genomics Institute, China), and bioinformatics analysis was also performed.

### Bioinformatics prediction and dual-luciferase reporter assay

Bioinformatics tools were applied to predict potential miR-98 binding sites of linc00665 (Starbase v2.0, FINDTAR3, and RegRNA2.0) and AKR1B10-3ʹ-UTR (mirDIP and microRNA.org). The putative miR-98 target binding sequences in linc00665 or AKR1B10-3ʹ-UTR and their mutant of the binding sites were synthesized and cloned downstream of the luciferase gene in the pmirGLO luciferase vector (Promega, USA). Cells were co-transfected with pmirGLO plasmid (pmirGLO-Linc00665-WT, pmirGLO-Linc00665-MUT, pmirGLO-AKR1B10-WT, pmirGLO-AKR1B10-MUT, or pmirGLO) and miR-98 mimics (or miR-NC). The luciferase activity was measured using the Dual-Luciferase Reporter Assay System (Promega, USA) 48 h after transfection. Firefly luciferase activity was normalized to Renilla luciferase activity.

### RNA pull down assay

Biotinylated linc00665 or control probes were synthesized by GenePharma (Shanghai, China), and incubated with Dynabeads M-280 Streptavidin (Invitrogen, CA, USA) according to the manufacturer’s protocols. The probe-coated beads were then incubated with A549 or H1299 cell lysates. The bounded RNA complexes were eluted and extracted for qRT-PCR analysis.

### Statistical analysis

Data were expressed as the mean ± standard deviation from at least three independent experiments. All statistical analyses were performed using IBM SPSS Statistics 22.0 or GraphPad Prism 7. Differences between groups were analyzed by Student’s *t*-test or nonparametric Mann–Whitney *U*-test. Pearson chi-square was used to analyze the associations between linc00665 expression and clinicopathological variables. Spearman correlation analysis was used for correlation between groups. Survival analysis was performed with the Kaplan–Meier method, and the log-rank test was used for comparisons. In addition, Cox proportional hazards model was used for univariate and multivariate analyses. A value of *p* < 0.05 was considered statistically significant.

## Results

### Identification of linc00665, which is upregulated in LUAD tissues

**Fig. 1:**
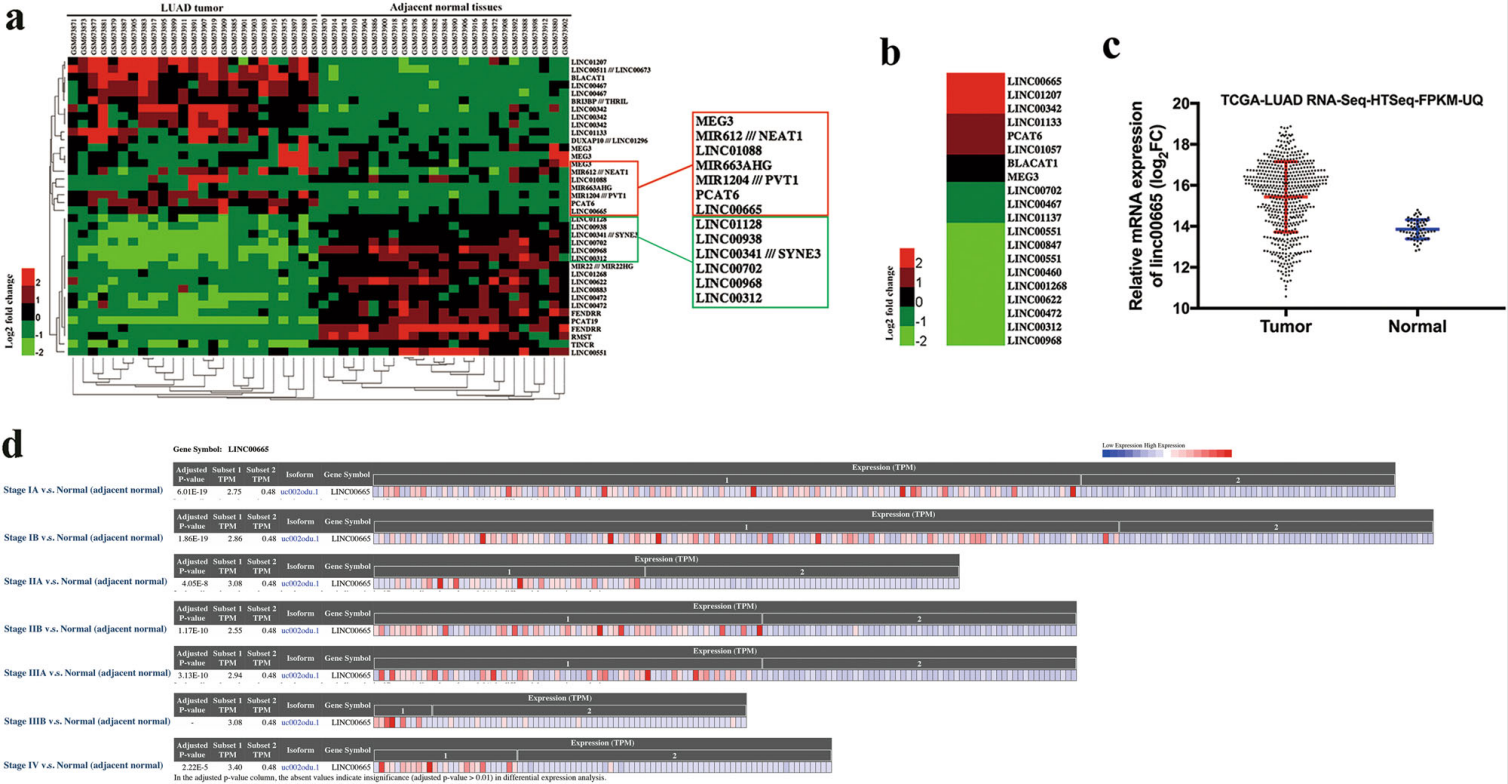
Identification of dysregulated lncRNAs in LUAD. **a** Cluster analysis of differentially expressed lncRNAs from microarray dataset GSE27262. **b** Relative expression levels of lncRNA candidates quantified by qRT-PCR in clinical samples. **c** Relative linc00665 expression in LUAD from TCGA database. In all, 533 LUAD tumor tissues and 59 normal tissues, respectively. **d** Linc00665 expression data from TCGA was analyzed by Cancer RNA-seq Nexus. LUAD lung adenocarcinoma, TCGA The Cancer Genome Atlas, qRT-PCR quantitative real-time PCR

To identify novel dysregulated lncRNAs in LUAD, a microarray dataset (GSE27262) from Gene Expression Omnibus was used to analyze differentially expressed lncRNAs between LUAD tumor samples and corresponding non-tumor samples, followed by validation in another independent microarray dataset (GSE19804). Cluster analysis showed a clear distinction between LUAD tumor and adjacent normal tissues (Fig. 1a). The expression levels of selected lncRNA candidates were then validated by qRT-PCR in 80 paired clinical LUAD tissues and corresponding normal tissues, among which linc00665 (ENST00000590622) was most significantly upregulated (6.3-fold, *p* < 0.001) in LUAD tissues (Fig. 1b). Consistently, analysis of TCGA-LUAD RNA-Seq dataset from The Cancer Genome Atlas (TCGA) showed that linc00665 was markedly upregulated in LUAD tissues compared with normal tissues (*p* < 0.0001, Fig. 1c). Further analysis of the LUAD RNA-seq dataset (572 samples) by using bioinformatics tool “Cancer RNA-seq Nexus” (http://syslab4.nchu.edu.tw/) indicated that expression levels of linc00665 were raised in all stages of LUAD tissues compared with adjacent normal tissues (Fig. 1d). What’s more, linc00665 was also hugely high expressed in lung squamous cell carcinoma, liver hepatocellular carcinoma, and breast invasive carcinoma (*p* < 0.0001), but low expressed in colon adenocarcinoma (*p* = 0.0006), compared with their adjacent normal tissues (supplementary Figure 1).

Information from the UCSC Genome Browser shows that linc00665 is a 1749-bp transcript with six exons and localizes in human chromosome 19q13.12. In addition, the Coding Potential Calculator (CPC) score of linc00665 was –1.02253, indicating no protein-coding potential for linc00665 (supplementary Figure 2a)^15^. Consistently, ORF Finder (National Center for Biotechnology Information) failed to predict a protein of >80 amino acids (supplementary Figure 2b), and the txCdsPredict score of linc00665 is 300.0, supporting that linc00665 has no protein-coding capacity.

### Linc00665 upregulation is associated with aggressive clinicopathological traits and poor prognosis for LUAD patients

**Fig. 2:**
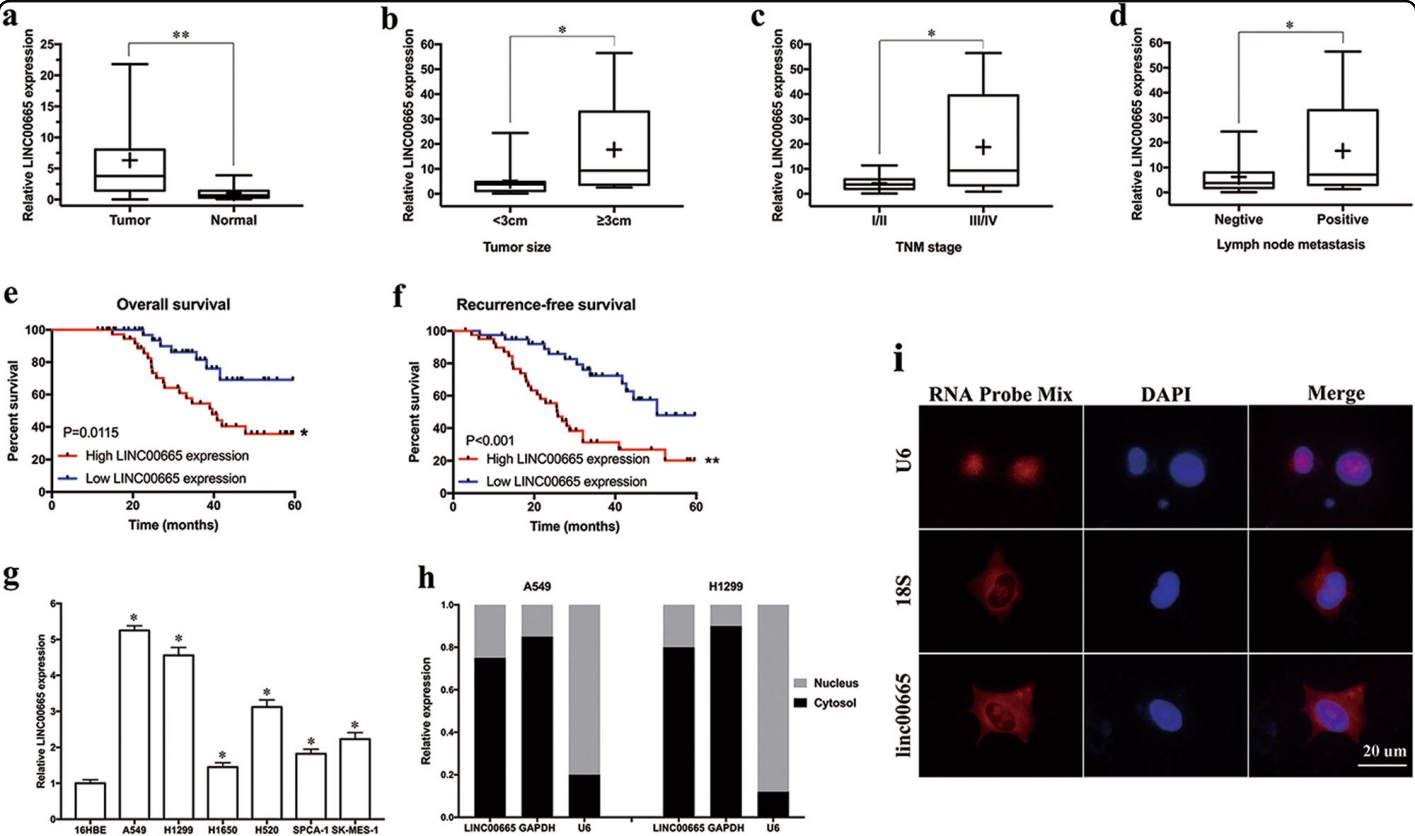
Expression of linc00665 in LUAD tissues and cells. **a** Linc00665 expression was significantly higher in LUAD specimens than in adjacent normal tissues. **b**–**d** Linc00665 expression was significantly higher in patients with big tumor size, advanced TNM stage and lymph node metastasis. Linc00665 expression was examined by qRT-PCR and normalized to GAPDH expression. **e**, **f** Kaplan–Meier survival analysis of overall and recurrence-free survival according to linc00665 expression in 80 LUAD patients. **g** Abundance of linc00665 in NSCLC cell lines relative to that in normal human bronchial epithelial cells (16HBE) as determined by qRT-PCR. **h** Subcellular location of linc00665 in A549 and H1299 cells. U6 and GAPDH acted as nucleus and cytoplasm marker, respectively. **i** RNA FISH assay to confirm subcellular location of linc00665 in A549 cells. U6 and 18S acted as nucleus and cytoplasm marker, respectively. LUAD lung adenocarcinoma, NSCLC non-small cell lung cancer, qRT-PCR quantitative real-time PCR; *p < 0.05, **p < 0.01

Expression levels of linc00665 were determined by qRT-PCR in 80 pairs of LUAD and adjacent normal tissues. The expression of linc00665 in tumor tissues was significantly higher than that in adjacent normal tissues (Fig. 2a). Accordingly, the patients were divided into two groups (relative high and low expression groups) according to the median level of linc00665 expression in LUAD tissues. Further analyses revealed that high linc00665 expression in LUAD tissues was remarkably corrected with larger tumor size (*p* = 0.0132), advanced TNM stage (*p* = 0.0363), and lymph node metastasis (*p* = 0.0066), but not correlated with other features such as age, gender, and differentiation (Table 1, Fig. 2b–d).

**Table 1:** Correlation between linc00665 expression and clinicopathological features in LUAD patients (*n* = 80)

Characteristics	Number (%)	Relative linc00665 expression		*p*-Value^a^
		Low	High	
Age (year)				
≤60	42 (52.5)	17	25	0.0733
>60	38 (47.5)	23	15	
Gender				
Male	51 (63.8)	29	22	0.1035
Female	29 (36.2)	11	18	
Differentiation				
Well moderate	45 (56.3)	26	19	0.1147
Poor	35 (43.7)	14	21	
Tumor size				
≤3 cm	35 (43.7)	23	12	0.0132*
>3 cm	45 (56.3)	17	28	
Lymph node metastasis				
Negative	46 (57.5)	29	17	0.0066*
Positive	34 (42.5)	11	23	
TNM stage				
I/II	51 (63.8)	30	21	0.0363*
III/IV	29 (36.2)	10	19	

LUAD lung adenocarcinoma

^*^P < 0.05

^a^Chi-square test

Kaplan–Meier survival curves indicated that patients with higher linc00665 expression had significantly reduced overall (*p* = 0.0115) and recurrence-free (*p* < 0.001) survival than did those with relative low linc00665 expression (Fig. 2e, f). Consistently, analysis of TCGA-LUAD datasets by using “Kaplan–Meier Plotter” (http://www.kmplot.com/analysis/index.php?p=service&cancer=lung) also showed that patients with high linc00665 expression in LUAD tissues suffered worse overall survival (*p* = 0.0085, supplementary Figure 3a). Moreover, multivariate Cox proportional hazard regression analysis identified positive lymph node metastasis, high TNM stage, and relative high linc00665 expression as independent prognostic factors for predicting poor recurrence-free survival in LUAD patients (Table 2).

**Table 2:** Univariate and multivariate analyses of factors associated with recurrence-free survival in LUAD patients

Variables	Univariate	Multivariate			
		Hazard ratio	95% CI		*p*-Value
Differentiation (poor vs. well moderate)	0.079				
Tumor size ( > 3 cm vs. ≤ 3 cm)	0.001	1.024		0.462–2.269	0.953
Lymph node metastasis (positive vs. negative)	<0.001	3.008		1.270–7.128	**0.012**
TNM stage (III/IV vs. I/II)	<0.001	2.393		1.012–5.659	**0.047**
Linc00665 expression (high vs. low)	<0.001	2.363		1.150–4.853	**0.019**

*LUAD* lung adenocarcinoma, *CI* confidence interval

Bold values indicate significant p values (<0.05)

Consistently, linc00665 was significantly higher expressed in six NSCLC cell lines than that in 16HBE cells (*p* < 0.05, respectively, Fig. 2g). A549 and H1299 cell lines with the highest linc00665 expression levels were selected for subsequent experiments. Subcellular fractionation assays implied that linc00665 was predominantly localized to the cytosol in both A549 and H1299 (Fig. 2h), which was confirmed by the RNA FISH assay in A549 cells (Fig. 2i). In conclusion, these data suggest that high linc00665 expression in tumor tissues represents a promising indicator of tumor progression and poor prognosis for LUAD.

### Linc00665 promotes LUAD cell proliferation, migration, invasion, and EMT in vitro

**Fig. 3:**
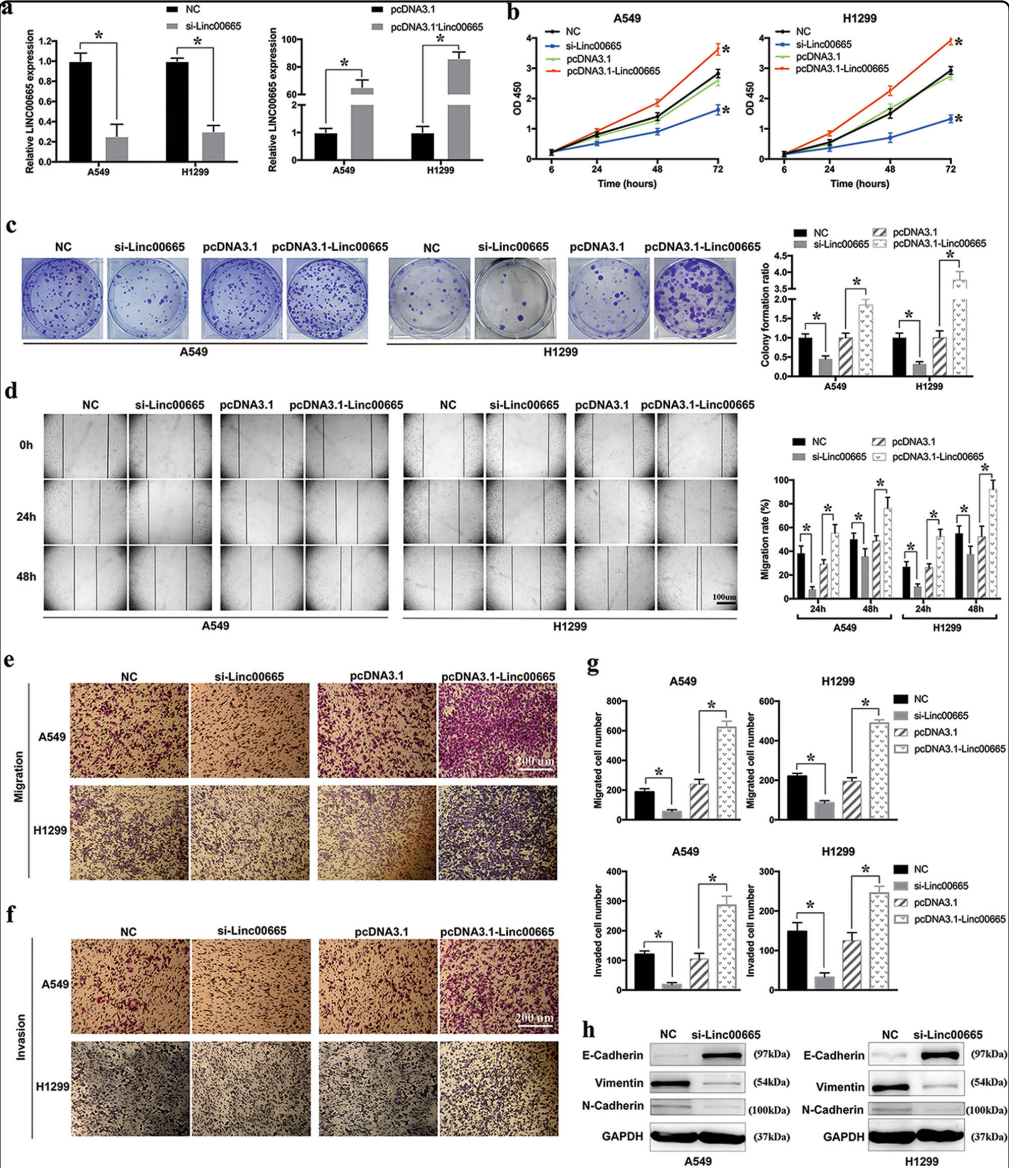
Effects of linc00665 on LUAD cell proliferation, migration, invasion, and EMT process in vitro. **a** Validation of siRNA knockdown and overexpression vector efficiency in A549 and H1299 cells as determined by qRT-PCR. **b** CCK-8 proliferation assays in A549 and H1299 cells after transfection with linc00665 siRNA or overexpression plasmid. **c** Representative images of colony formation assays in A549 and H1299 cells after transfection, with the media changed and siRNA transfection repeated every 3 days. **d** Representative images of wound-healing assays at indicated times after scratching in A549 and H1299 cells. **e**, **f** Representative images of transwell migration and invasion assays in A549 and H1299 cells after transfection. **g** Quantified bar charts of migrated or invaded cell numbers. **h** Expression of E-cadherin, Vimentin, N-cadherin and GAPDH protein in A549 and H1299 cells after transfection with linc00665 siRNA. LUAD lung adenocarcinoma, NC negative control, qRT-PCR quantitative real-time PCR; *p < 0.05

To investigate the roles of linc00665 in LUAD, loss- and gain-of-function experiments were performed. In both A549 and H1299 cell lines, siRNA-mediated knockdown and plasmid-mediated overexpression were conducted for manipulating linc00665 expression, which was validated by qRT-PCR (Fig. 3a). To avoid the “off target” effect, three linc00665 siRNAs with knockdown efficiency over 50% were used in functional assays and similar results were collected. Representative images of loss-of-function experiments showed in Fig. 3 were obtained from linc00665 siRNA (UCCUCAGUCUUGGGCUAUUTT).

Functionally, CCK-8 assays showed that linc00665 knockdown significantly inhibited vitality of A549 and H1299 cells, whereas linc00665 overexpression promoted cell proliferation in comparison with that of their counterparts (Fig. 3b). Similarly, colony formation assays revealed that linc00665 knockdown caused a remarkable decrease in clonogenic survival of A549 and H1299 cells, and that linc00665 overexpression exhibited a significant increase in the clonogenic survival (Fig. 3c). Notably, the proliferation promoted by linc00665 overexpression in CCK-8 and colony formation assays could be reversed by subsequent linc00665 knockdown in A549 and H1299 cells (supplementary Figure 4). What’s more, linc00665 overexpression significantly promoted cell proliferation in H1650 LUAD cells, whereas no substantial difference was found in 16HBE bronchial epithelial cells (supplementary Figure 5a, b). Furthermore, wound-healing assays and transwell migration assays indicated that linc00665 knockdown markedly suppressed the migration ability of A549 and H1299 cells, whereas linc00665 overexpression facilitated cell migration (Fig. 3d, e, g). Moreover, transwell invasion assays demonstrated that linc00665 knockdown repressed the invasion of A549 and H1299 cells, and linc00665 overexpression had opposite effects on cell invasion (Fig. 3f, g). Besides, A549 and H1299 cells pre-transfected with linc00665 siRNA or overexpression plasmid were seeded for 24-h CCK-8 assays. The results showed that proliferation of cells in different conditions revealed no significant difference during the first 24 h, compared with their negative controls, indicating that the observed impact of linc00665 in migration or invasion is not due to its influence in cell proliferation (supplementary Figure 5c). Together, these results implied that linc00665 could promote LUAD cell proliferation, migration and invasion in vitro.

Epithelial–mesenchymal transition (EMT) progression plays a critical part in cancer cell migration and invasion. Therefore, we explored the role of linc00665 in EMT of LUAD cells. Western blot data showed that linc00665 knockdown significantly elevated the expression of epithelial marker E-cadherin and decreased the levels of mesenchymal marker Vimentin and N-cadherin in A549 and H1299 cells (Fig. 3h). The results indicated that linc00665 promoted EMT to enhance migration and invasion of LUAD cells.

### Knockdown of linc00665 promotes cell cycle arrest and induces cell apoptosis in vitro

**Fig. 4:**
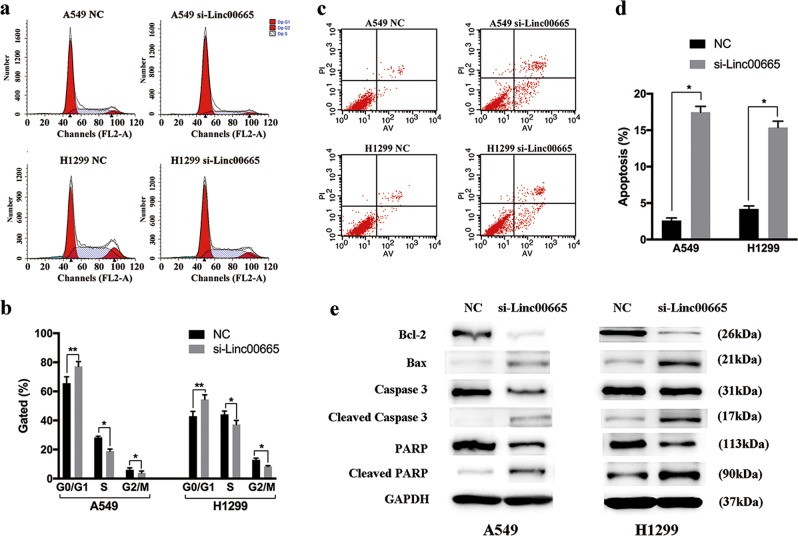
Knockdown of linc00665 promotes cell cycle arrest and induces cell apoptosis in vitro. **a**, **b** Flow cytometric analysis of cell cycle distributions in A549 and H1299 cells after transfection with linc00665 siRNA. **c**, **d** Flow cytometric analysis of apoptosis in A549 and H1299 cells after transfection. **e** Expression of Bcl-2, Bax, Caspase-3, PARP, and GAPDH protein in A549 and H1299 cells after transfection with linc00665 siRNA. NC negative control; *p < 0.05, **p < 0.01

Flow cytometry was then performed to analyze the impact of linc00665 on cell cycle distributions and apoptosis. Linc00665 knockdown in A549 and H1299 cells induced cell cycle arrest at G0/G1 phase and a decrease of cells at S phase compared with negative control (Fig. 4a, b). Meanwhile, the proportion of apoptotic cells was significantly increased following linc00665 knockdown in A549 and H1299 cells (Fig. 4c, d). Consistently, expression levels of apoptosis-related proteins, including Bax, Cleaved Caspase-3, and Cleaved poly ADP-ribose polymerase (PARP), were markedly increased in linc00665 knockdown A549 and H1299 cells, whereas Bcl-2 expression was significantly diminished compared with negative controls (Fig. 4e). Collectively, these results showed that linc00665 knockdown led to promotion of the proportion of G0/G1 phase and cell apoptosis.

### Knockdown of linc00665 suppresses LUAD cell proliferation and metastasis in vivo

**Fig. 5:**
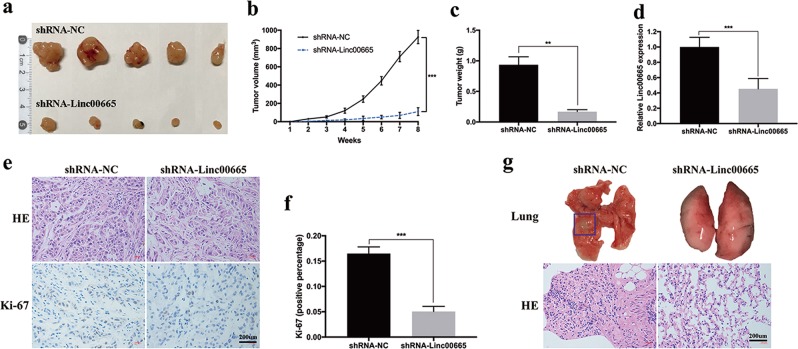
Downregulation of linc00665 suppressed LUAD tumor growth and metastasis in vivo. **a** Representative images of tumors collected from mice. **b** Tumor volume curve of mice upon shRNA-NC or shRNA-Linc00665 treatment. **c** Tumor weights were represented. **d** Relative linc00665 expression in xenograft tumors was detected by qRT-PCR, normalized to GAPDH. **e** Histopathology of xenograft tumors. The tumor sections were under HE staining and immunohistochemical staining using antibodies against Ki-67. **f** Ki-67 index calculated as the percentage of Ki-67-positive cells. **g** Representative images of lung metastasis and HE staining of sections. The metastasis was marked with box. Scale bar = 200 μm; NC negative control, HE hematoxylin and eosin, qRT-PCR quantitative real-time PCR; **p < 0.05, ***p < 0.01

To further confirm the oncogenic role of linc00665 in vivo, xenograft mouse models were applied. Knockdown linc00665 dramatically decreased tumor growth in vivo, which was determined by significantly reduced tumor size and weight compared with negative controls (Fig. 5a–c). In addition, the mean expression of linc00665 in xenograft tumors generated from linc00665-silenced A549 cells was much lower than that in tumors from control cells (Fig. 5d). Moreover, typical characteristics of the tumors were revealed by hematoxylin and eosin (HE) staining, and tumor cell proliferation was evaluated using Ki-67 immunohistochemical staining. As shown, positive Ki-67 staining was significantly attenuated in linc00665-silenced tumors relative to controls (Fig. 5e, f). Notably, metastatic lung nodes were observed in 4 of the 10 xenograft mice treated with A549/shRNA-NC, whereas none metastatic lung node was found in linc00665-silenced groups (Fig. 5g). The difference was statistically significant (chi-square = 5.0, *p* = 0.0253). Taken together, these data implied that knockdown of linc00665 inhibited tumor growth and metastasis in vivo.

### Linc00665 promotes LUAD progression by upregulating downstream target AKR1B10

**Fig. 6:**
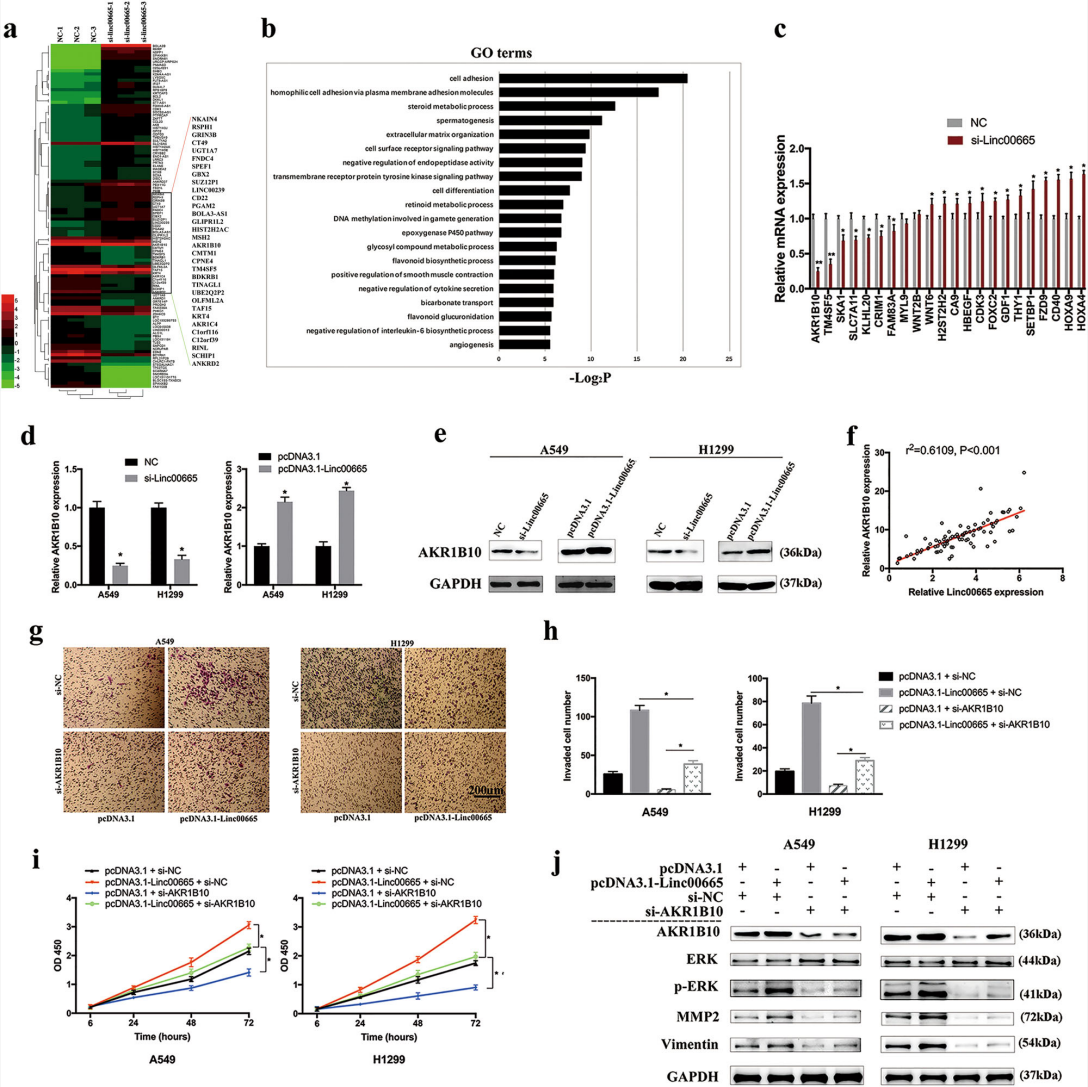
Identification of AKR1B10 as a downstream target of linc00665 in LUAD cells. **a** RNA transcriptome sequencing analysis was performed to analyze gene expression profiling in A549 cells following linc00665 knockdown. **b** Gene ontology analysis for all genes with altered expressions. **c** The altered mRNA levels of genes were selectively confirmed by qRT-PCR in A549 cells following linc00665 knockdown. **d** mRNA level of AKR1B10 was confirmed by qRT-PCR in A549 and H1299 cells following linc00665 knockdown or overexpression. **e** Protein level of AKR1B10 was confirmed by western blot in A549 and H1299 cells following linc00665 knockdown or overexpression. **f** Correlation between linc00665 and AKR1B10 expression in 80 LUAD samples. **g**, **h** Representative images and quantification of transwell invasion assays in A549 and H1299 cells after transfection with linc00665 overexpression plasmid or AKR1B10 siRNA. **i** CCK-8 proliferation assays in A549 and H1299 cells after transfection with linc00665 overexpression plasmid or AKR1B10 siRNA. **j** Expression of AKR1B10, ERK, p-ERK, MMP2, Vimentin, and GAPDH protein in A549 and H1299 cells after transfection with linc00665 overexpression plasmid or AKR1B10 siRNA. Original uncropped western images are shown in Supplementary Figure 7. NC negative control, LUAD, lung adenocarcinoma, qRT-PCR quantitative real-time PCR; *p < 0.05, **p < 0.01

To assess linc00665-associated gene expression profiles in LUAD, RNA transcriptome sequencing was carried out with linc00665 knockdown A549 cells and control cells. A set of 479 differentially expressed mRNAs (|log2(FoldChange)| > 1.5 and *p* < 0.05) were recognized and gene oncology analysis by DAVID system showed that the most significantly overrepresented biological processes included cell adhesion, extracellular matrix organization, cell differentiation, cytokine secretion, interleukin-6 biosynthesis, and angiogenesis (Fig. 6a, b). Accordingly, expression of selective oncogenes or tumor suppressor genes was examined by qRT-PCR in linc00665 knockdown A549 cells and control cells. The results determined that AKR1B10 was the most downregulated in response to linc00665 downregulation in A549 cells (Fig. 6c).

Furthermore, linc00665 knockdown significantly decreased the expression of AKR1B10 in both A549 and H1299 cells, whereas linc00665 overexpression markedly elevated AKR1B10 expression levels, in both mRNA and protein levels, which was confirmed by qRT-PCR and western blot (Fig. 6d, e). Consistently, linc00665 expression was positively correlated with AKR1B10 in 80 clinical LUAD tissues (*p* < 0.001, *r*^2^ = 0.6109; Fig. 6f). Whereas, no significant correlation was found between AKR1B10 and linc00665 expression in 80 clinical normal lung tissues (supplementary Figure 6a), which was consistent with the data from TCGA RNA-seq datasets (supplementary Figure 6b). Functionally, cell proliferation and invasion were significantly inhibited by AKR1B10 downregulation in A549 and H1299 cells, and AKR1B10 knockdown dramatically abolished the role of linc00665 in promoting cell proliferation and invasion (Fig. 6g–i). These data suggested that AKR1B10 was responsible for the oncogenic role of linc00665 in LUAD. Additionally, it has previously been reported that overexpressed AKR1B10 in LUAD was a significant prognostic factor for poor recurrence-free survival^16^. Analysis of TCGA survival data revealed that high AKR1B10 expression in LUAD was correlated with poor overall survival (supplementary Figure 3b) and that the cohorts with synchronously high expressions of linc00665 and AKR1B10 in LUAD tissues had the worst 5-year overall survival (*p* = 0.005, supplementary Figure 3c). Thus, the positive association between linc00665 and AKR1B10 expression in LUAD correlates with patients’ poor outcomes.

It has been reported that AKR1B10 stimulates breast cancer cell migration and invasion by activating ERK signaling^17^. To address whether AKR1B10-ERK signaling was involved in the function of linc00665, expressions of ERK, p-ERK, matrix metalloproteinase-2 (MMP2), and vimentin were detected by western blot (Fig. 6j). The results indicated that p-ERK1/2, MMP2 and vimentin were upregulated in linc00665 overexpressed cells, whereas their expression levels were decreased in AKR1B10 knockdown cells. Furthermore, the elevated expression of p-ERK1/2, MMP2, and vimentin caused by linc00665 overexpression could be reversed by AKR1B10 inhibition. ERK signaling pathway has been well documented in the regulation of cell growth and differentiation, and improperly activation contributes to malignant transformation^18^. Taken together, this indicates that AKR1B10-mediated ERK signal pathway might lead to the oncogenic function of linc00665 in A549 and H1299 cells.

### Linc00665 functions as a molecular sponge of miR-98, liberating AKR1B10 mRNA transcripts

**Fig. 7:**
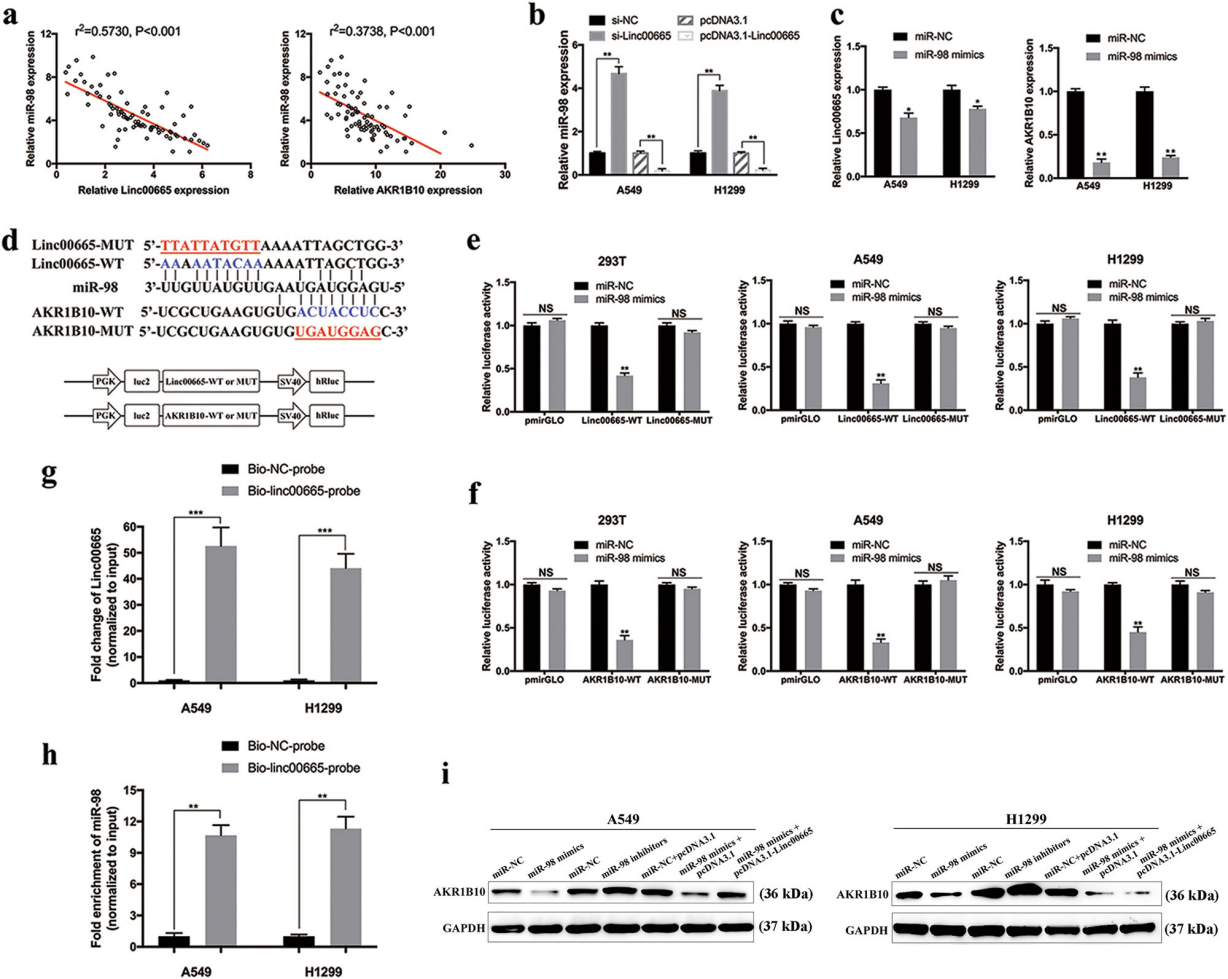
Linc00665 upregulates AKR1B10 by competitively binding to miR-98. **a** Correlation between miR-98 and linc00665 or AKR1B10 expression in 80 LUAD samples. **b** Relative miR-98 expression in A549 and H1299 cells following linc00665 knockdown or overexpression. **c** Relative linc00665 or AKR1B10 expression in A549 and H1299 cells after transfection with miR-98 mimics. **d** Putative binding sites of miR-98 to linc00665/AKR1B10, and schematic of wild-type and mutant pmirGLO-linc00665/pmirGLO-AKR1B10 constructs. **e** miR-98 mimics or mimics NC and pmirGLO-linc00665-WT or pmirGLO-linc00665-MUT were co-transfected into 293T, A549, and H1299 cells. Luciferase activity was detected 24 h after transfection using the dual-luciferase assay. **f** miR-98 mimics or mimics NC and pmirGLO-AKR1B10-WT or pmirGLO-AKR1B10-MUT were co-transfected into 293T, A549, and H1299 cells. Luciferase activity was detected 24 h after transfection using the dual-luciferase assay. **g**, **h** Biotin-labeled RNA pull down assays were performed to confirm the binding of linc00665 with miR-98 in A549 and H1299 cells. A significant amount of linc00665 and miR-98 were observed in linc00665-probe pulled down pellets. **i** Expression of AKR1B10 and GAPDH protein in A549 and H1299 cells after transfection with miR-98 mimics, miR-98 inhibitors, or linc00665 overexpression plasmid. NC negative control, NS not significant, LUAD, lung adenocarcinoma; *p < 0.05, **p < 0.01, ***p<0.001.

LncRNAs may function as a ceRNA through binding miRNAs, thereby de-repressing their target mRNA transcripts^7–10^. Multiple bioinformatics databases predicted that both linc00665 and AKR1B10 sequences contained putative binding sites of miR-98 (Fig. 7d). To clarify their potential interactions, expressions of linc00665, miR-98, and AKR1B10 were analyzed in LUAD tissues by qRT-PCR. Spearman correlation analysis showed that the expression of miR-98 was negatively correlated with the expression of linc00665 (*r*^2^ = 0.573, *p* < 0.001) and AKR1B10 (*r*^2^ = 0.3738, *p* < 0.001) (Fig. 7a). In A549 and H1299 cells, linc00665 knockdown enhanced miR-98 expression, whereas linc00665 overexpression significantly reduced miR-98 levels (Fig. 7b). Moreover, treatment with miR-98 mimics significantly inhibited mRNA levels of linc00665 and AKR1B10 in A549 and H1299 cells (Fig. 7c). Also, the proliferation promoted by linc00665 overexpression in CCK-8 could be significantly reverted by miR-98 mimics in A549 and H1299 cells (supplementary Figure 5d).

To further validate the binding of miR-98, dual-luciferase assays were conducted in 293T, A549, and H1299 cells. Luciferase vectors were constructed as described previously (Fig. 7d). Cells co-transfected with miR-98 mimics and the wild-type linc00665 vector (pmirGLO-Linc00665-WT), but not the mutant linc00665 reporter vector (pmirGLO-Linc00665-MUT), exhibited significantly decreased luciferase activity (Fig. 7e). Similarly, miR-98 significantly reduced the luciferase activity of wild-type AKR1B10 3ʹ-UTR reporter, whereas no significant effect was observed when the putative binding site was mutated (Fig. 7f). Biotin-labeled RNA pull down assays were performed to further confirm that linc00665 is physically associated with miR-98 in A549 and H1299 cells (Fig. 7g, h). A significant amount of linc00665 and miR-98 were observed in linc00665-probe pulled down pellets, compared with control groups (*p* < 0.01, respectively). These data suggested that miR-98 could directly target linc00665 and AKR1B10.

In addition, the regulatory relationships among linc00665, miR-98, and AKR1B10 were further analyzed by western blot (Fig. 7i). Treatment with miR-98 mimics sharply diminished protein levels of AKR1B10 in A549 and H1299 cells, whereas miR-98 inhibitors markedly raised AKR1B10 expression. Moreover, linc00665 overexpression partly reversed the inhibiting effect of miR-98 mimics on AKR1B10 in A549 cells (*p* < 0.05), whereas no significant differences in AKR1B10 protein levels were observed in H1299 cells (*p* > 0.05). In brief, the results indicated that linc00665 functions as a ceRNA through binding miR-98, thereby de-repressing the expression of AKR1B10 in LUAD cells.

### SP1 upregulates the transcription of linc00665

**Fig. 8:**
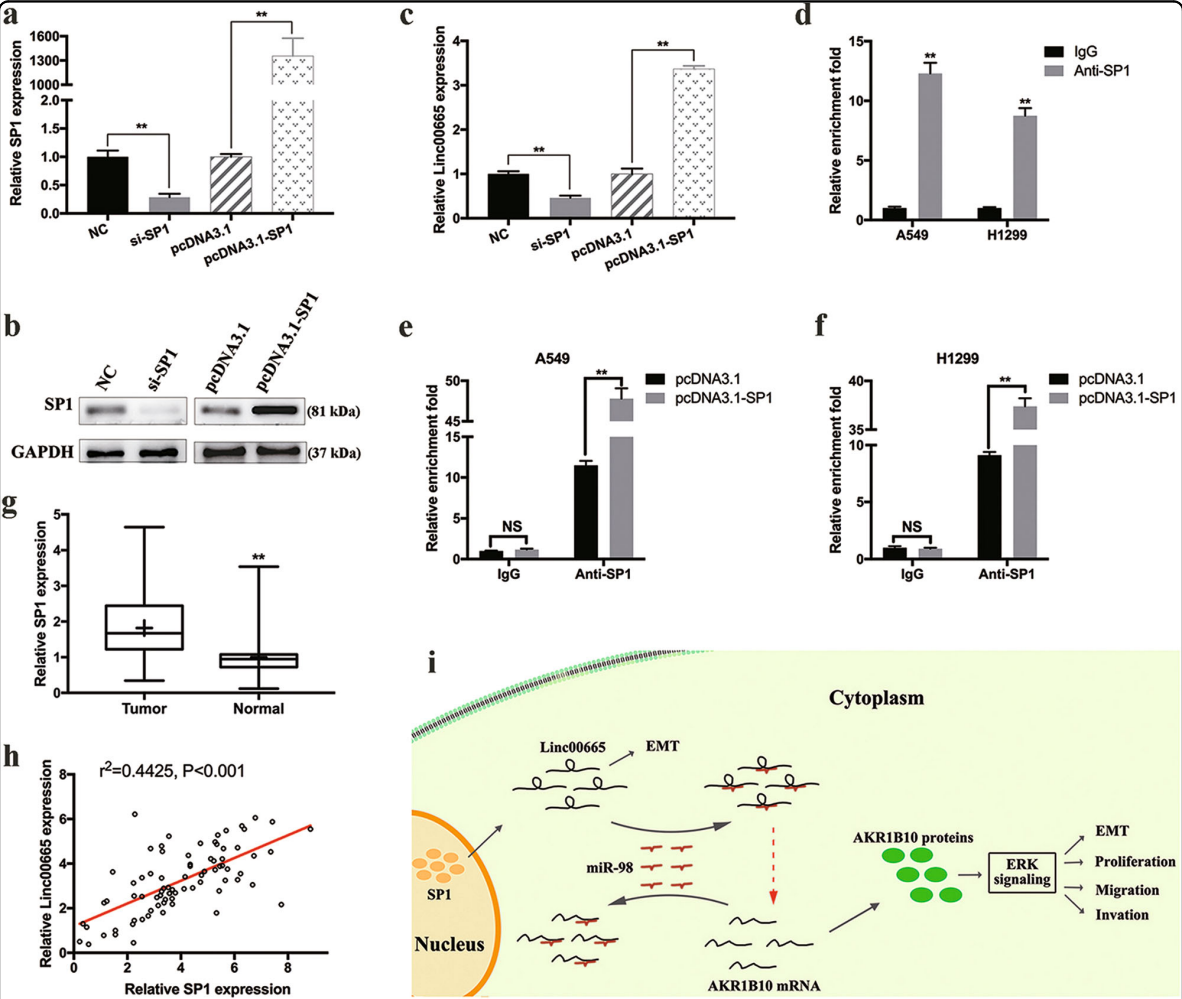
Expression of linc00665 was upregulated by transcription factor SP1. **a**, **b** Validation of SP1 siRNA knockdown and overexpression vector efficiency in A549 cells as determined by qRT-PCR and western Blot. **c** Relative linc00665 expression in A549 cells after transfected with SP1 siRNA or overexpression plasmid. **d** ChIP assay was used to detect the binding of SP1 protein to linc00665 promoter in A549 and H1299 cells. **e**, **f** The sequence –42 to –33-bp upstream of linc00665 was detected in SP1 immunoprecipitates, which was further enhanced upon overexpressing SP1 in A549 and H1299 cells, as determined by ChIP assay. **g** Expression levels of SP1 mRNA in the 80 paired LUAD tissues by qRT-PCR. **h** Correlation between SP1 and linc00665 expression in 80 LUAD samples. **i** Schematic diagram of linc00665-based regulatory mechanism in LUAD cells. NC negative control, NS not significant, LUAD, lung adenocarcinoma, ChIP chromatin immunoprecipitation, qRT-PCR quantitative real-time PCR; **p < 0.01

Bioinformatics tools JASPAR (http://jaspar.genereg.net/) and PROMO (http://alggen.lsi.upc.es/) were applied to predict transcription factors binding to linc00665 promoter. One putative SP1 binding site located 42–33-bp upstream of linc00665 was identified. As a general transcription factor, SP1 is widely expressed in mammalian cells, and SP1‐dependent transcription is highly regulated throughout development, cellular differentiation, and tumorigenesis^19^. SP1 targeted siRNA and overexpression plasmid were constructed, and the efficiency was validated in A549 cells by qRT-PCR and western blot (Fig. 8a, b). In A549 cells, the expression of linc00665 was noticeably attenuated upon SP1 knockdown (*p* < 0.01), and substantially increased upon SP1 overexpression (*p* < 0.01) (Fig. 8c). ChIP assays were then performed, and the sequence –42 to –33-bp upstream of linc00665 was detected in SP1 immunoprecipitates (Fig. 8d), which was further enhanced upon overexpressing SP1 in A549 and H1299 cells (Fig. 8e, f). Moreover, SP1 expression was significantly elevated (1.82 ± 0.17-folds, *p* < 0.01) in 80 LUAD tissues, compared with that in corresponding normal tissues (Fig. 8g), and the expression of linc00665 was positively correlated with that of SP1 in clinical LUAD tissues (*r*^2^ = 0.4425, *p* < 0.001, Fig. 8h). In addition, RNA­seq datasets from TCGA were analyzed and the results suggested a significantly positive correlation between SP1 expression and that of linc00665 in liver hepatocellular carcinoma (*r*^2^ = 0.0212, *p* = 0.0050), but not in lung squamous cell carcinoma, breast invasive carcinoma, or colon adenocarcinoma (supplementary Figure 6c). Taken together, transcription factor SP1 binds to the promoter of linc00665 and partly upregulates the transcription of linc00665 in LUAD cells.

## Discussion

Mounting evidences have suggested the notion that lncRNAs play critical roles in tumorigenesis of multiple cancers, whereas only few of them have been well functionally characterized^5^. In this study, we reported the identification of a novel lncRNA linc00665, which was highly expressed in LUAD tissues compared with adjacent normal tissues. The expression of linc00665 in LUAD tumor tissues was closely associated with patients’ aggressive clinicopathological characteristics and could serve as an independent predictor of recurrence-free survival. A recent microarray analysis focusing on expression profiles of lncRNAs in LUAD listed linc00665 as part of upregulated lncRNAs^20^. Bioinformatics analysis using public databases also suggested that linc00665 overexpression in hepatocellular carcinoma might facilitate cancer progression by regulating cell cycle pathways^21^. Consistently, the data analysis from Cancer RNA-seq Nexus inducing TCGA database showed that linc00665 was significantly upregulated in all stages of LUAD tissues. Accordingly, we proposed oncogenic roles for linc00665 in LUAD.

With loss- and gain-of-function assays, we further demonstrated that linc00665 promoted LUAD cell proliferation, migration, invasion, and EMT process in vitro, as well as modulating cell cycle arrest and apoptosis. In vivo assays showed that linc00665 could boost tumor growth and metastasis in xenograft models. Abundant studies have highlighted the importance of EMT in driving tumor cell local invasion and systemic dissemination. During EMT process, cells lose their epithelial characteristics and undergo transition into mesenchymal phenotypes, such as adherens junction and apical-basal polarity^22^. Our results showed that inhibition of linc00665 increased the expression of epithelial markers and decreased mesenchymal markers, indicating that effects of linc00665 on cell migration and invasion were partly associated with EMT process.

As summarized in Fig. 8i, we confirmed the hypothesis that transcription factor SP1 induced the transcription of linc00665, which exerted its oncogenic role by acting as a ceRNA for miR-98 and subsequently activating the AKR1B10-ERK signaling pathway. Being structurally similar to mRNA, lncRNAs can be directly targeted by microRNAs, acting as ceRNA to regulate mRNAs at a post-transcriptional level, thereby modulating tumor development^7–10^. In this study, we found that miR-98 was negatively correlated with linc00665 and AKR1B10 in LUAD tissues. Bioinformatics analysis, luciferase reporter assays, and rescue experiments defined that miR-98 targeted both linc00665 and AKR1B10. It was previously predicted that linc00665 was targeted by 26 microRNAs in co-expression network, suggesting that linc00665 might play a role as a sponge to indirectly de-repress a series of mRNAs in nasopharyngeal carcinoma^23^. Besides, a MYC-miR98-linc00665 feed-forward loop was computationally revealed in breast cancer^24^. Additionally, miR-98 was previously reported to act as a tumor suppressor in NSCLC, and low serum miR-98 might be an unfavorable prognostic biomarker for NSCLC patients^25,26^. Conversely, AKR1B10 has been shown to be overexpressed in multiple cancers, particularly in NSCLC, promoting tumor growth and progression^16,17,27,28^. In breast cancer, AKR1B10 promoted cancer cell migration and invasion through activation of ERK signaling and upregulation of MMP2 and vimentin, which was in accordance with our results^17^. The ERK signaling pathway promoted tumor cell invasion and cancer metastasis through activation of MMPs, including MMP2 especially, contributing to the degradation of extracellular matrix^29,30^.

As shown above, lncRNAs may participate the regulation of numerous targets using diverse binding regions, and one gene can also be regulated by multiple lncRNAs. Thus, the regulatory network of lncRNAs could be much complicated in cells. Although we demonstrated the interaction between linc00665 and miR98–AKR1B10–ERK signaling, there might be some additional pathways involved in the regulatory circuits of linc00665 in LUAD. Therefore, further studies should be conducted to deeply investigate the regulatory networks in cancer. Moreover, Northern blot assays, as well as 5ʹ and 3ʹ Rapid amplification of cDNA ends (RACE), should be performed in following studies to determine whether the major or full transcript of linc00665 in vivo is in line with that from UCSC.

In summary, we reported oncogenic roles for linc00665 in LUAD. Our results indicated that linc00665 was overexpressed in LUAD tissues and associated with poor prognosis. Moreover, linc00665 reinforced LUAD cell proliferation and invasion in vitro and in vivo, through functioning as a ceRNA for miR-98 and subsequently activating the AKR1B10–ERK signaling pathway, therefore emphasizing the potentials of this axis in LUAD diagnosis and therapy.

## Supplementary information

Supplementary Figure 1

Supplementary Figure 2

Supplementary Figure 3

Supplementary Figure 4

Supplementary Figure 5

Supplementary Figure 6

Supplementary Figure 7

Supplementary Table 1

Supplementary Table 2

Supplemental figure legends
