# Supplementary figures and images for: Long non-coding RNA linc00665 promotes lung adenocarcinoma progression and functions as ceRNA to regulate AKR1B10-ERK signaling by sponging miR-98
Source: Cell Death Dis. 2019 Jan 28;10(2):84. doi: 10.1038/s41419-019-1361-3 (PMC6349882; doi:10.1038/s41419-019-1361-3)

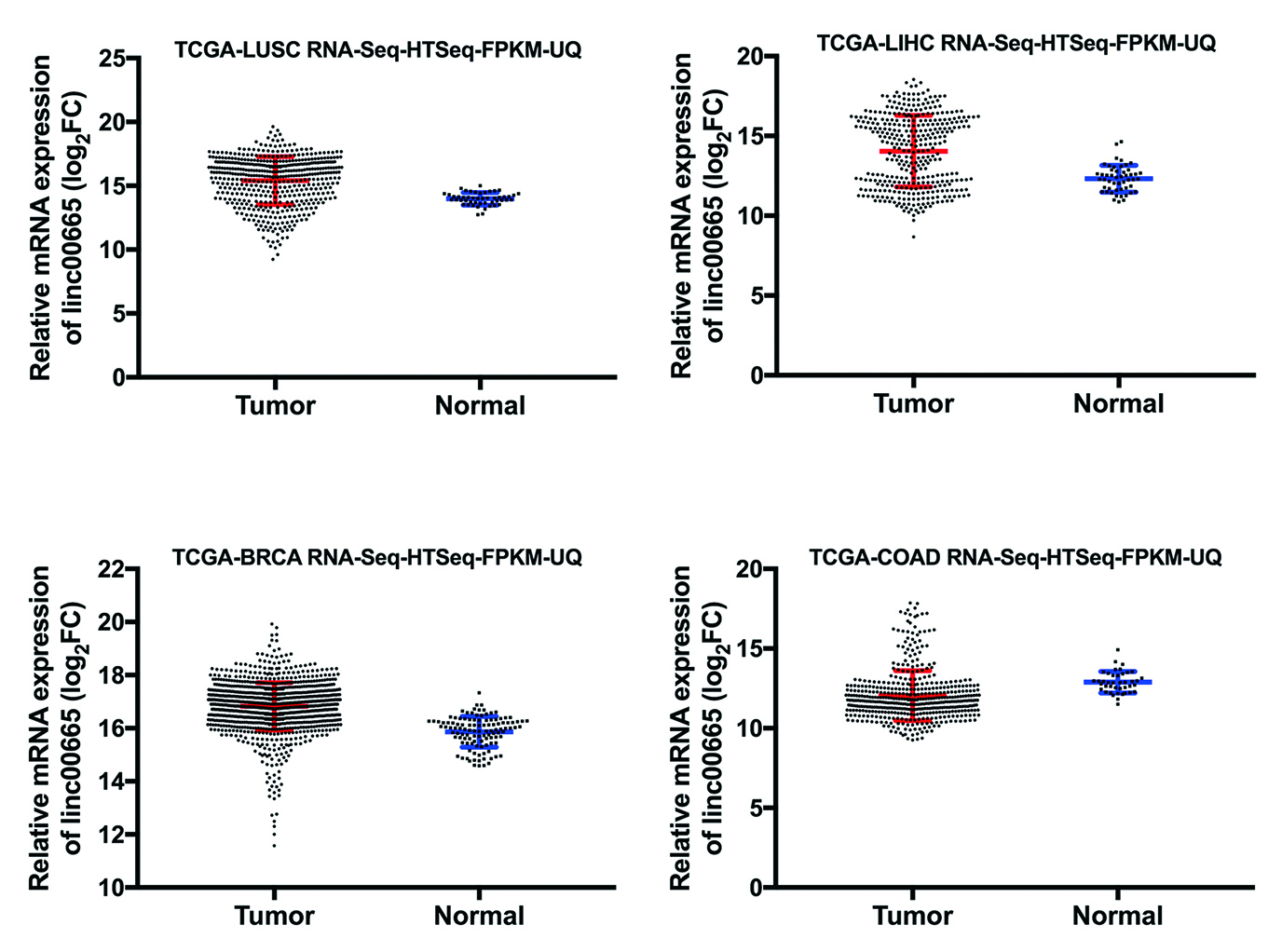

Supplement: Supplementary file 1 — Supplementary Figure 1 [file 41419_2019_1361_MOESM1_ESM.jpg]

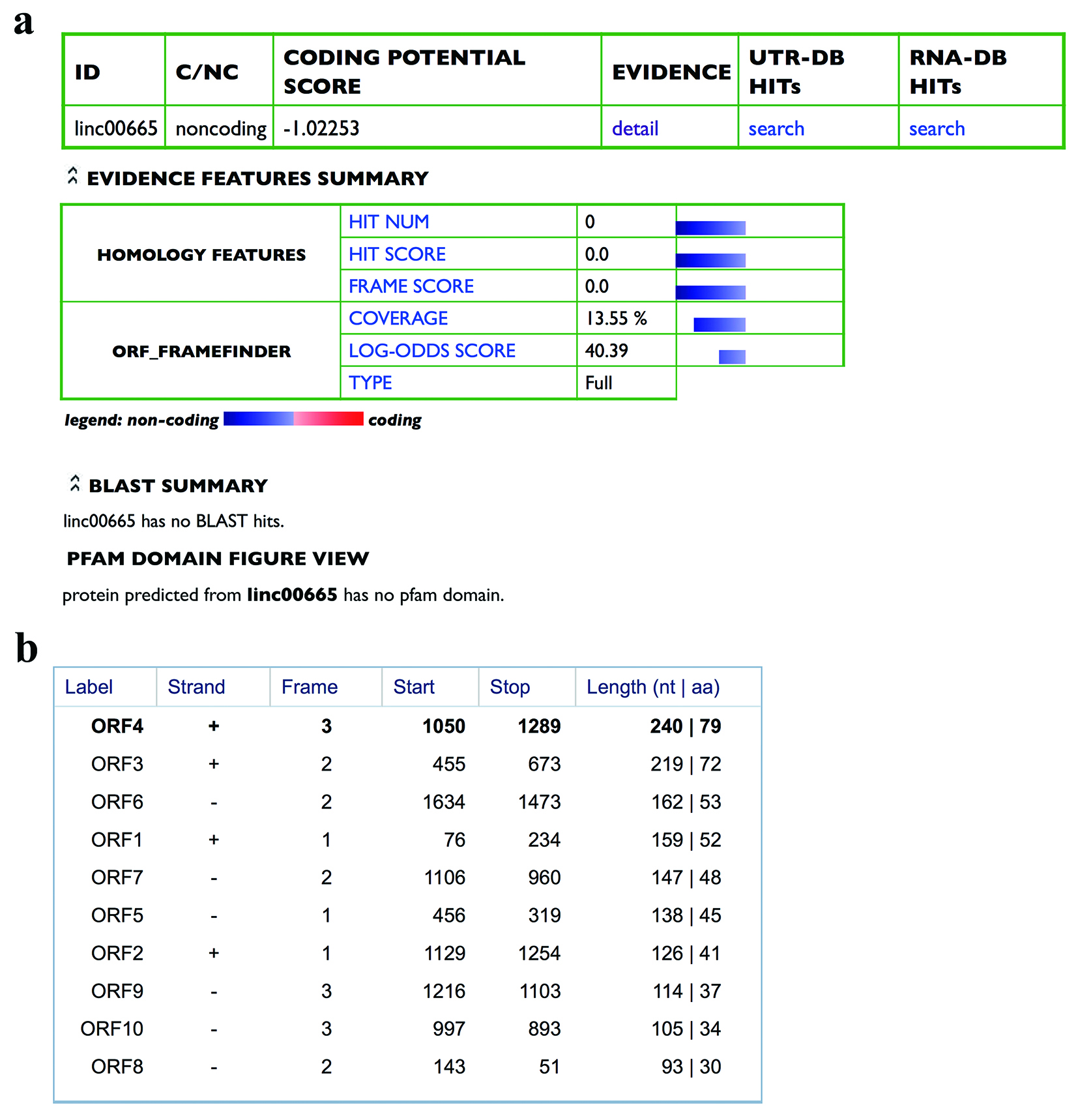

Supplement: Supplementary file 2 — Supplementary Figure 2 [file 41419_2019_1361_MOESM2_ESM.jpg]

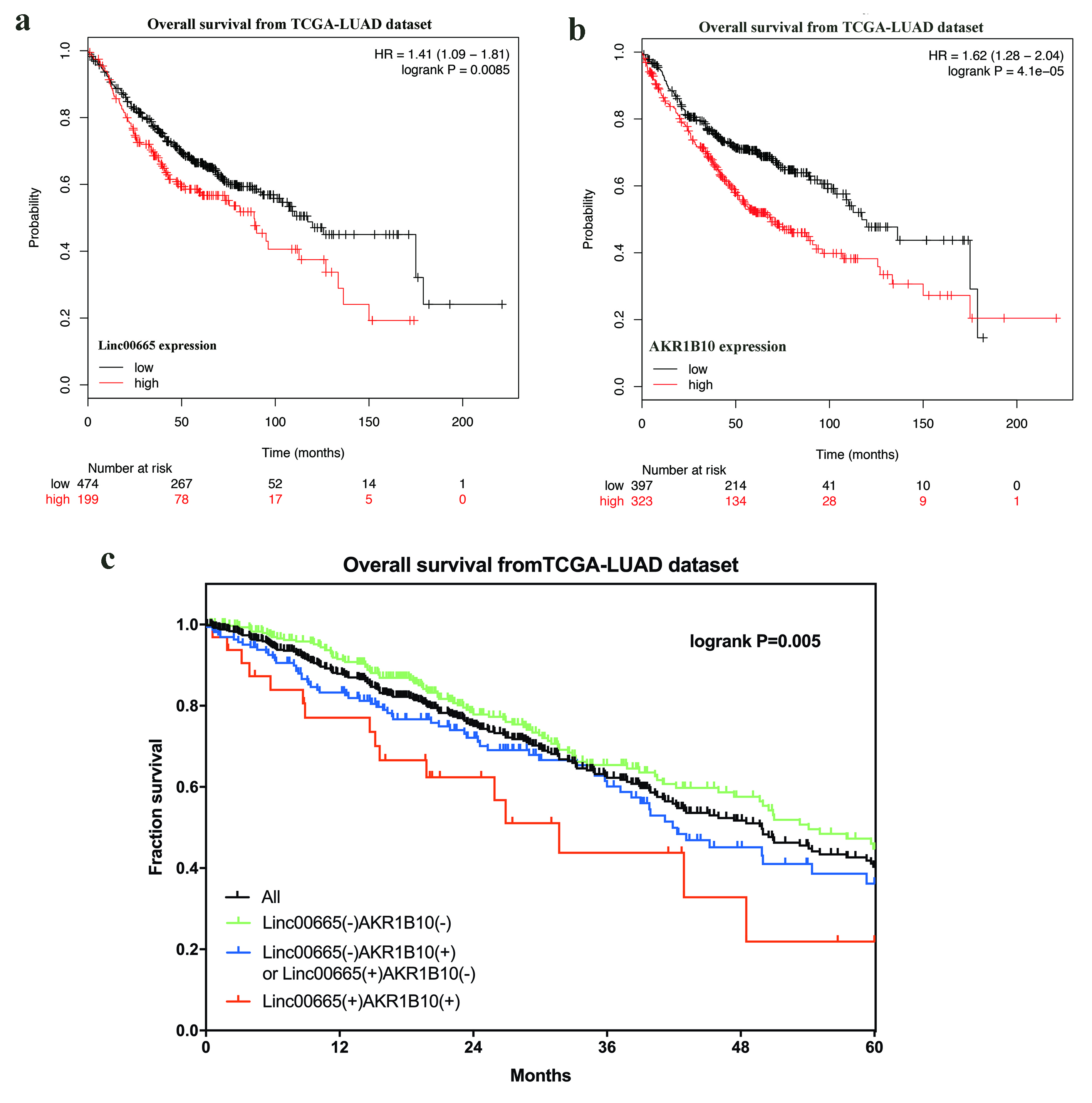

Supplement: Supplementary file 3 — Supplementary Figure 3 [file 41419_2019_1361_MOESM3_ESM.jpg]

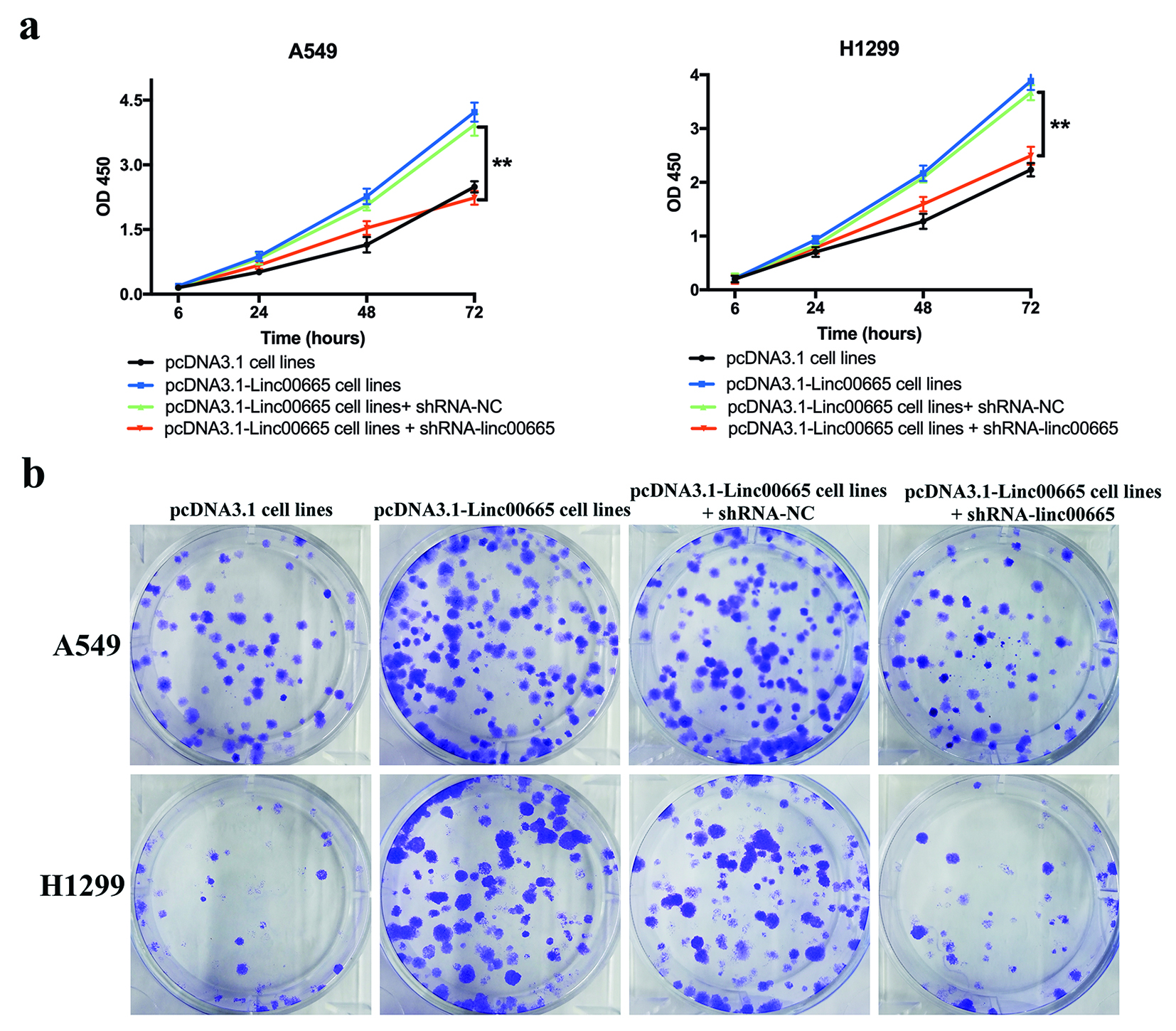

Supplement: Supplementary file 4 — Supplementary Figure 4 [file 41419_2019_1361_MOESM4_ESM.jpg]

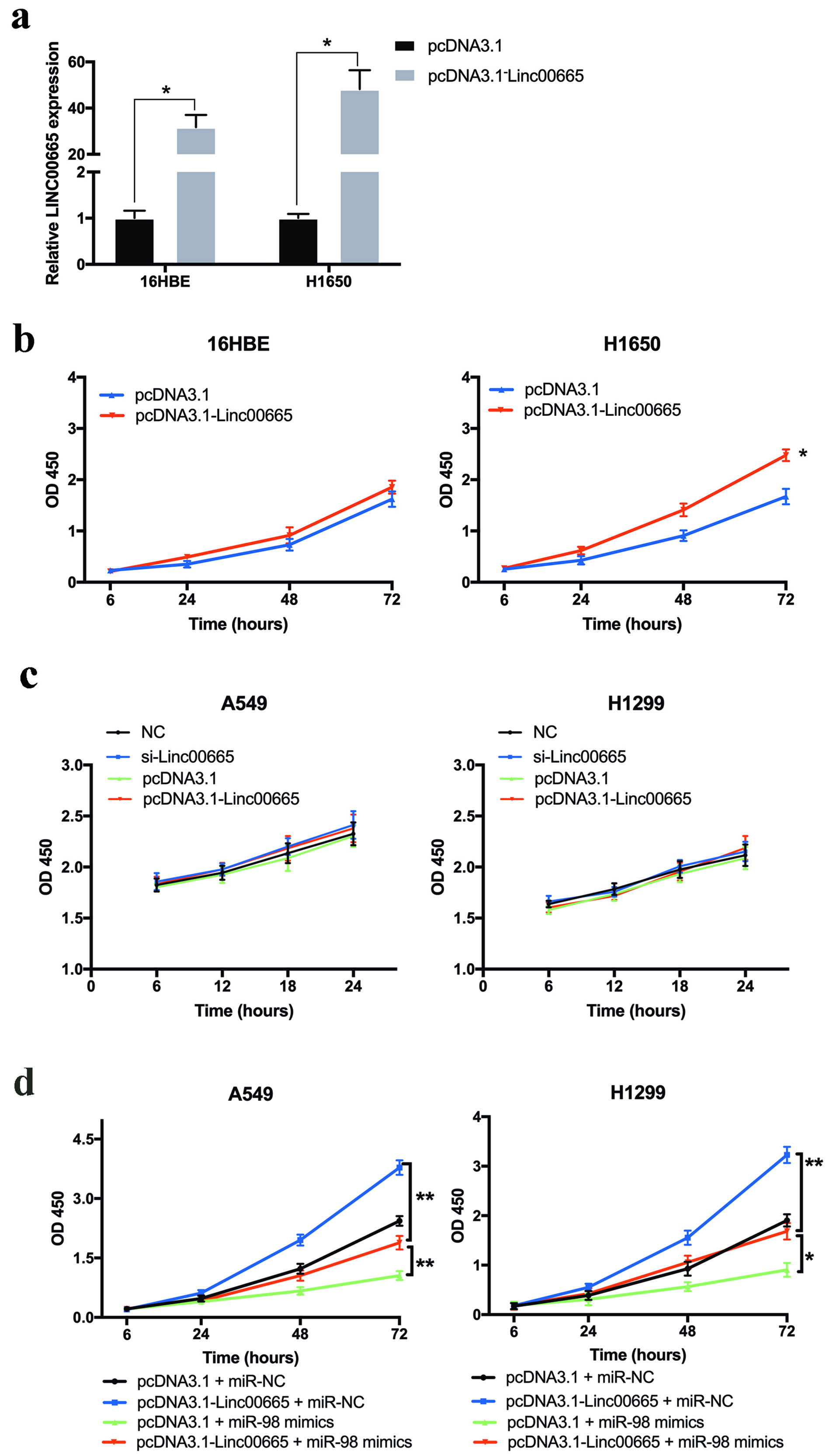

Supplement: Supplementary file 5 — Supplementary Figure 5 [file 41419_2019_1361_MOESM5_ESM.jpg]

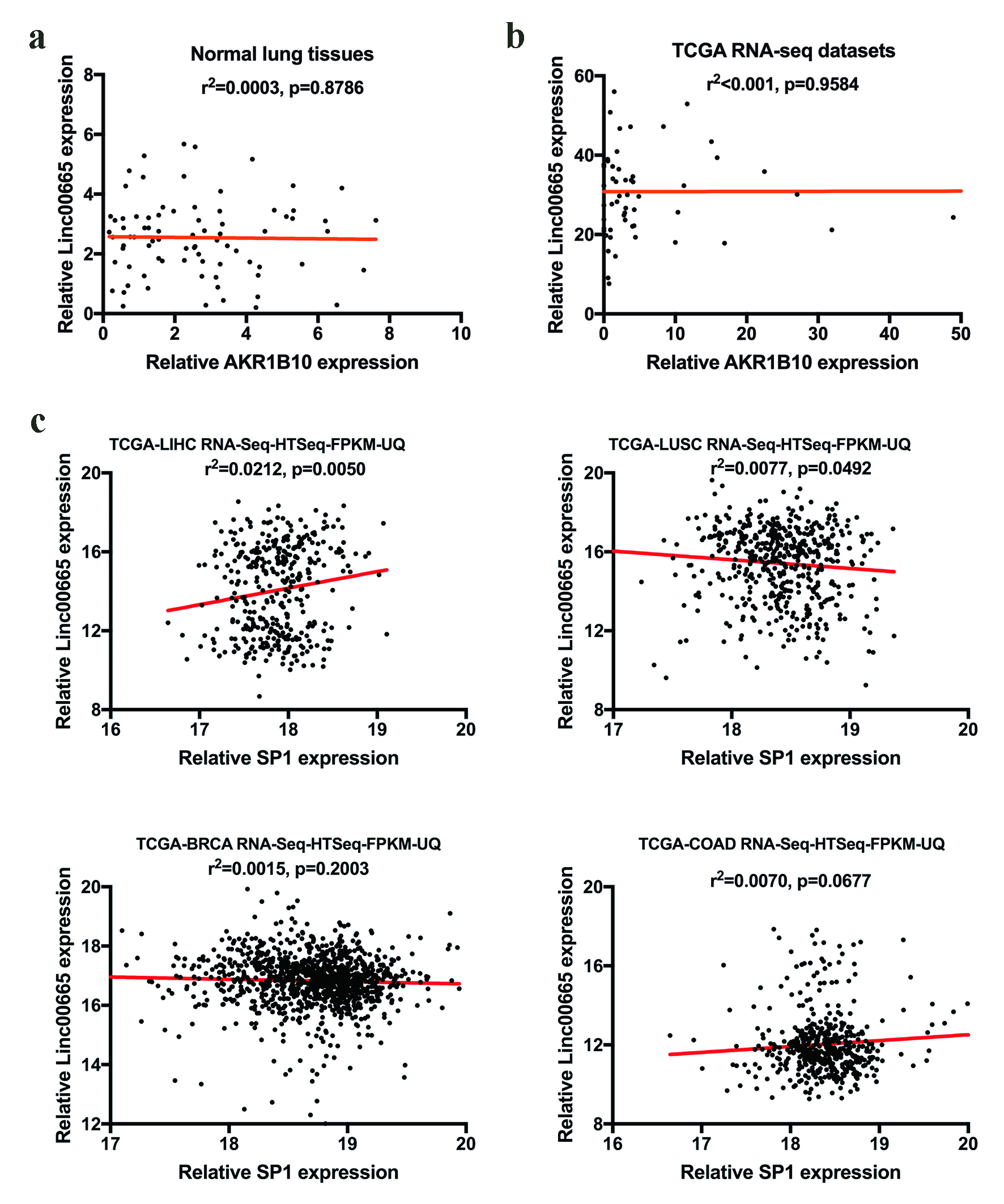

Supplement: Supplementary file 6 — Supplementary Figure 6 [file 41419_2019_1361_MOESM6_ESM.jpg]

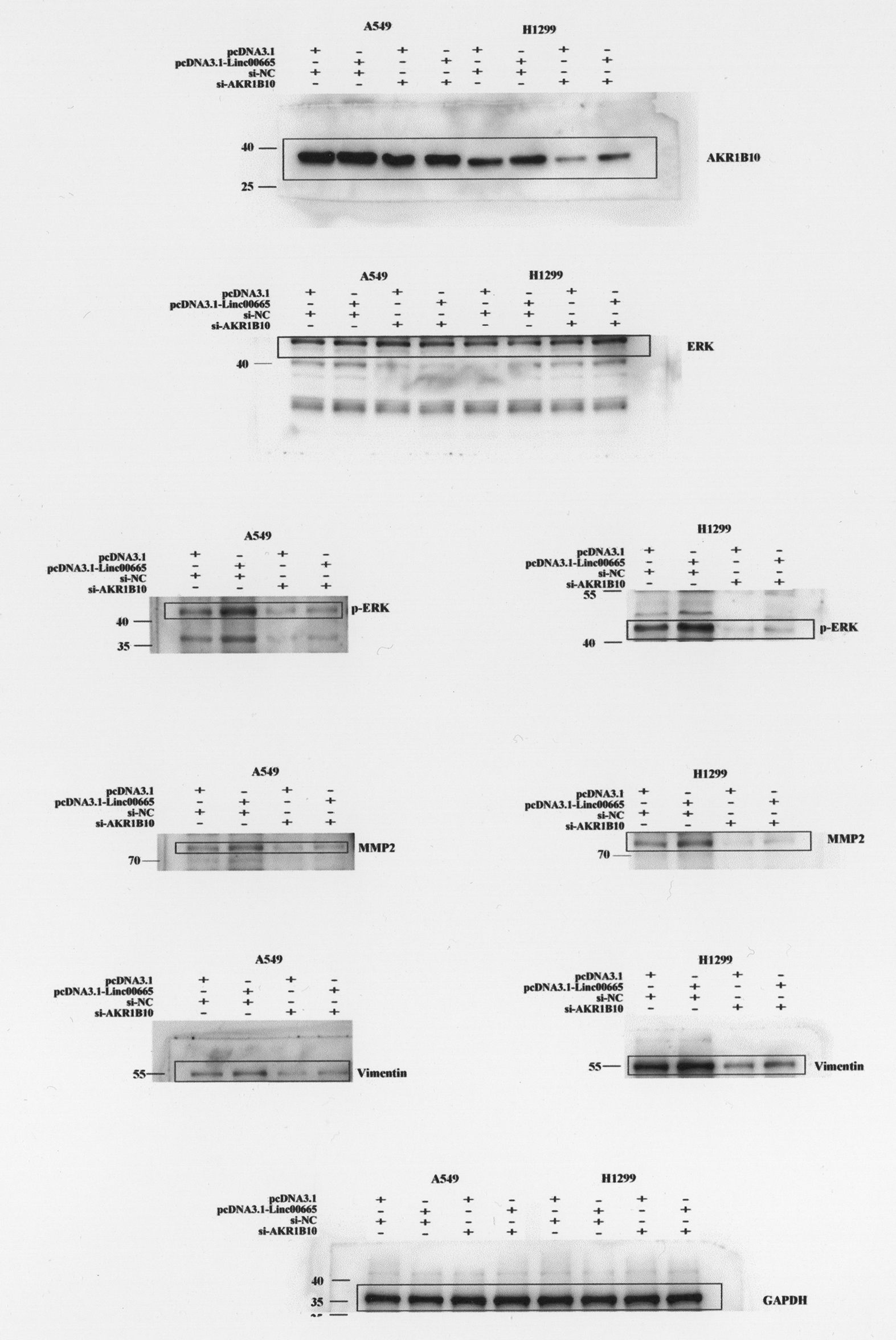

Supplement: Supplementary file 7 — Supplementary Figure 7 [file 41419_2019_1361_MOESM7_ESM.jpg]
