# Supplementary material for: Long non-coding RNA linc00665 promotes lung adenocarcinoma progression and functions as ceRNA to regulate AKR1B10-ERK signaling by sponging miR-98
Source: Cell Death Dis. 2019 Jan 28;10(2):84. doi: 10.1038/s41419-019-1361-3 (PMC6349882; doi:10.1038/s41419-019-1361-3)
Supplement: Supplementary file 8 — Supplementary Table 1 [file 41419_2019_1361_MOESM8_ESM.docx]

**Supplementary Table 1. Sequences of siRNA and shRNA**

| **Name** | **Sequence** |
| --- | --- |
| si-NC | 5'-UUCUCCGAACGUGUCACGUTT-3' |
| si-Linc00665 | 5'-UCCUCAGUCUUGGGCUAUUTT-3' |
| si-AKR1B10 | 5'-CGAGAAUCGAGGUGCUGUUTT-3' |
| si-SP1 | 5'-GGUAGCUCUAAGUUUUGAUTT-3' |
| shRNA-NC | 5'-CCGGCAACAAGATGAAGAGCACCAACTCGAGTTGGTGCTCTTCATCTTGTTGTTTTT-3' |
| shRNA-Linc00665 | 5'-CACCGTCCTCAGTCTTGGGCTATTTTCGAAAAAATAGCCCAAGACTGAGGA-3' |

NC, negative control.
