# Supplementary material for: Long non-coding RNA linc00665 promotes lung adenocarcinoma progression and functions as ceRNA to regulate AKR1B10-ERK signaling by sponging miR-98
Source: Cell Death Dis. 2019 Jan 28;10(2):84. doi: 10.1038/s41419-019-1361-3 (PMC6349882; doi:10.1038/s41419-019-1361-3)
Supplement: Supplementary file 9 — Supplementary Table 2 [file 41419_2019_1361_MOESM9_ESM.docx]

**Supplementary Table 2. Primers for qRT-PCR**

| **Gene** | **Primers** |
| --- | --- |
| GAPDH | Forward 5′-GCAAATTCCATGGCACCGT-3′ |
|  | Reverse 5′-TCGCCCCACTTGATTTTGG-3′ |
| U6 | Forward 5’-CTCGCTTCGGCAGCACA-3’ |
|  | Reverse 5’-AACGCTTCACGAATTTGCGT-3’ |
| Linc00665 | Forward 5'-CTGGCGCTGATGTAGTTT-3' |
|  | Reverse 5'-TTTTTCAGGTGGCACTCC-3' |
| AKR1B10 | Forward 5'-TCAGAATGAACATGAAGTGGGG-3' |
|  | Reverse 5'-TGGGCCACAACTTGCTGAC-3' |
| SP1 | Forward 5'-AGTTCCAGACCGTTGATGGG-3' |
|  | Reverse 5'-GTTTGCACCTGGTATGATCTGT-3' |
