# Supplementary material for: Long non-coding RNA linc00665 promotes lung adenocarcinoma progression and functions as ceRNA to regulate AKR1B10-ERK signaling by sponging miR-98
Source: Cell Death Dis. 2019 Jan 28;10(2):84. doi: 10.1038/s41419-019-1361-3 (PMC6349882; doi:10.1038/s41419-019-1361-3)
Supplement: Supplementary file 10 — Supplemental figure legends [file 41419_2019_1361_MOESM10_ESM.docx]

**Supplementary Figure 1.** Relative linc00665 expression in LUSC, LIHC, BRCA and COAD from TCGA database. LUAD, lung adenocarcinoma; LUSC, lung squamous cell carcinoma; LIHC, liver hepatocellular carcinoma; BRCA, breast invasive carcinoma; COAD, colon adenocarcinoma; TCGA, The Cancer Genome Atlas.

**Supplementary Figure 2.** Protein-coding potential of linc00665 predicted by (a) Coding Potential Calculator and (b) ORF Finder.

**Supplementary Figure 3.** Overall survival in LUAD from TCGA datasets. (a) Analysis of overall survival in LUAD from TCGA by using “Kaplan-Meier Plotter”, based on linc00665 expressions. (b) Analysis of overall survival in LUAD from TCGA by using “Kaplan-Meier Plotter”, based on AKR1B10 expressions. (c) Overall survival in LUAD from TCGA datasets, according to relative linc00665 and AKR1B10 mRNA expressions. LUAD, lung adenocarcinoma; TCGA, The Cancer Genome Atlas.

**Supplementary Figure 4.** Rescue experiments in A549 and H1299 cells with manipulated linc00665 expression. Linc00665-overexpression cell lines were further transfected with linc00665-targeted or control shRNA. (a) CCK-8 proliferation assays. (b) Representative images of colony formation assays.

**Supplementary Figure 5.** CCK-8 proliferation assays. (a) Expression of linc00665 in 16HBE and H1650 cells, validated by qRT-PCR. (b) CCK-8 proliferation assays in 16HBE and H1650 cells. (c) 24-hours CCK-8 assays in A549 and H1299 cells. Cells were seeded at a density of 2.5x10^4^/well (the density approximates to that in the upper chamber for migration assays). (d) CCK-8 assays in A549 and H1299 cells, by mimics transfection into linc00665-overexpressed cell lines.

**Supplementary Figure 6.** (a) Correlation between AKR1B10 and linc00665 expression in 80 clinical normal lung specimens. (b) Correlation between AKR1B10 and linc00665 expression in normal lung tissues from TCGA datasets. (c) Correlation between the expression levels of linc00665 and SP1 in LIHC, LUSC, BRCA and COAD from TCGA database. LUSC, lung squamous cell carcinoma; LIHC, liver hepatocellular carcinoma; BRCA, breast invasive carcinoma; COAD, colon adenocarcinoma; TCGA, The Cancer Genome Atlas.

**Supplementary Figure 7.** Original uncropped western blot images for Figure 6j.

**Supplementary Table 1.** Sequences of siRNA and shRNA

**Supplementary Table 2.** Primers for qRT-PCR
